# Assessment of the Quality and Evaluation of the Antioxidant Potential of a Novel Sri Lankan Ayurvedic Polyherbal Formulation

**DOI:** 10.1155/2020/2319315

**Published:** 2020-07-17

**Authors:** H. P. Wakkumbura, W. M. D. Wickramaarachchi, L. D. A. M. Arawwawala, J. A. Liyanage, R. P. V. J. Rajapakse

**Affiliations:** ^1^Department of Kaumarabruthya and Stree Roga, Gampaha Wickramarachchi Ayurveda Institute, University of Kelaniya, Yakkala, Sri Lanka; ^2^Department of Veterinary Pathobiology, Faculty of Veterinary Medicine and Animal Science, University of Peradeniya, Sri Lanka; ^3^Industrial Technology Institute, Bauddhaloka Mawatha, Colombo 07, Sri Lanka; ^4^Department of Chemistry, Faculty of Science, University of Kelaniya, Yakkala, Sri Lanka

## Abstract

**Background:**

In Sri Lanka, a Polyherbal Ayurvedic Formulation (PHAF), which consists of powders of seven medicinal plants, is being trialed for use as an anti-inflammatory drug. In general, anti-inflammatory drugs have good antioxidant properties. Therefore, in the present study, an attempt was made to assess the quality and evaluate the antioxidant potential of PHAF.

**Methods:**

Physicochemical parameters such as ash content, extractable matter, phytochemical screening for secondary metabolites, levels of heavy metals, and microbes were determined according to standard protocols. Antioxidant activity was evaluated using five in vitro assays: total polyphenolic content (TPC), total flavonoid content (TFC), ORAC (oxygen radical absorbance capacity), DPPH (1,1-diphenyl-2-picryl-hydrazyl), and ABTS (2,2-azino-bis(3-ethylbenzothiazoline-6-sulfonic acid) diammonium salt.

**Results:**

PHAF contained 5.6 ± 0.2% of moisture, 6.5 ± 0.1% of total ash, 1.4 ± 0.1% of water soluble ash, 0.9 ± 0.0% of acid insoluble ash, 7.7 ± 0.2% of hot water extractable matter, 3.9 ± 0.1% of cold water extractable matter, 10.5 ± 4.1% of hot-ethanol extractable matter, and 8.4 ± 0.2% of cold-ethanol extractable matter. Phytochemical screening revealed the presence of phenolic compounds, tannins, flavonoids, coumarins, and saponins in both aqueous and ethanolic extracts of the drug. TPC, TFC, ORAC, DPPH, and ABTS of aqueous and ethanol extracts of PHAF were 103.65 ± 4.94 and 327.07 ± 9.65 mg gallic acid equivalents/g extract, 76.6 ± 5.83 and 224.6 ± 8.42 mg quercetin equivalents/g of extract, 481.11 ± 17.30 and 1481.44 ± 30.20 mg trolox equivalents/g of extract, 79.50 ± 4.42 and 227.17 ± 6.16 mg trolox equivalents/g of extract, and 198.20 ± 4.55 and 577.08 ± 5.48 mg trolox equivalents/g of extract, respectively.

**Conclusion:**

Ethanolic extract of PHAF is better than aqueous extract in terms of antioxidant properties.

## 1. Introduction

Since prehistoric times, herbs were the basis for nearly all medicinal therapy until synthetic drugs were developed in the nineteenth century, a time when the prominence of herbal remedies gradually decreased in developed countries [1, 2]. Interest in herbal medicine has increased recently, with their efficacy again being researched and identified. Plant-based compounds and formulations for treatment of various ailments are becoming common place in society. Moreover, 75–80% of the world's population in developing countries continues to use herbal medicines for primary healthcare. However, quality assessments of herbal medicines are frequently lacking, something urgently required for the safety of users.

Free radicals are molecular species or atoms which contain one or more unpaired electrons. These are constantly produced in the human body as a result of cell metabolism [3]. Free radicals have important roles in gene expression and activation of receptors as well as in regulation of signal transduction [4]. However, an excess of free radicals can become toxic to living cells [5] and may cause many diseases such as cancer, atherosclerosis, neurodegenerative diseases, and inflammation [6–8].

Previous researches have indicated that phenolic compounds and flavonoids act as excellent anti-inflammatory agents [9]. Due to the relation of these compounds towards antioxidant activity, it is important to evaluate the antioxidant potential.

A novel Polyherbal Ayurvedic Formulation (PHAF), which consists of powders of seven medicinal plants (Table 1), is being trialed for use as an anti-inflammatory drug in Sri Lanka. As PHAF has shown promising anti-inflammatory activity in initial studies, in the present study, an attempt was done to assess the quality and evaluate the antioxidant potential of the drug.

## 2. Materials and Methods

### 2.1. Plant Ingredients

Fresh plant materials required for PHAF were collected from Colombo (6° 55′ 54.98″ N *x* 79° 50′ 52.01″ E) Western province, Sri Lanka, and authenticated by the Curator of National Herbarium of Peradeniya, Sri Lanka. The plant materials were identified and authenticated by Prof. M. H. A. Tissera, Professor in Dravyaguna, Department of Dravyaguna, Gampaha Wickramarachchi Ayurveda Institute, University of Kelaniya, Yakkala, Sri Lanka. A voucher specimen from each plant (PHAF1-7) was deposited at Department of Kaumarabruthya and Stree Roga, Gampaha Wickramarachchi Ayurveda Institute, University of Kelaniya, Yakkala, Sri Lanka, for future reference.

### 2.2. Preparation of Polyherbal Ayurvedic Formulation (PHAF)

In brief, all the ingredients were washed thoroughly and air dried. Then ingredients were pulverized separately into a coarse powder and combined in a stainless steel vessel in a ratio of 1 : 1 (w/w) and mixed well.

### 2.3. Preparation of Hot-Ethanol Extract from Polyherbal Ayurvedic Formulation (PHAF)

PHAF powder (50 g) was added to a round bottom flask containing 150 mL of ethanol and boiled for 4 h. Then, the extract was filtered using Whatman 0.45 *μ*m filter paper, and the filtrate was concentrated using a rotary evaporator (yield: 9.2% w/w) and stored at 4°C until used.

### 2.4. Preparation of Hot-Water Extract from Polyherbal Ayurvedic Formulation (PHAF)

PHAF powder (50 g) was added to a round bottom flask containing 150 mL of water and boiled for 4 h. Then, the extract was filtered using Whatman 0.45 *μ*m filter paper and the filtrate was concentrated using a rotary evaporator and freeze dried (yield: 12.5% w/w) and stored at 4°C until used.

### 2.5. Physicochemical Investigation

Physicochemical parameters of PHAF powder were assayed according to the World Health Organization (WHO) guidelines on quality control methods for herbal materials [10]. Physicochemical parameters such as water extractable matter (both hot and cold), ethanol extractable matter (both hot and cold), total ash, water soluble ash, acid insoluble ash, and moisture content were investigated.

### 2.6. Qualitative Phytochemical Screening

Phytochemical screening was carried out for PHAF (ethanol and aqueous extracts) according to methods described by Yadav and Agarwala [11] with slight modifications.

### 2.7. Determination of Heavy Metal Content

Quantitative determination of arsenic (As) [12], mercury (Hg) [13], cadmium (Cd) [13], and lead (Pb) [12] was carried out according to the Association of Official Agricultural Chemists (AOAC) methods using an Inductively Coupled Plasma Mass Spectrometry.

### 2.8. Determination of Microbial Limits

Microbial tests for aerobic plate count [14], coliform count [15], yeasts and moulds count [16], *Escherichia coli* [17]*, Salmonella* [18], and *Staphylococcus aureus* [19] were carried out as per standard procedures detailed in Sri Lanka Standards.

### 2.9. Evaluation of Antioxidant Activity

Antioxidant activity of the ethanol and aqueous extracts of PHAF powder was investigated using the following in vitro assays.

#### 2.9.1. Total Polyphenolic Content (TPC)

Total polyphenol content of ethanol and aqueous extracts of PHAF was determined using the Folin–Ciocalteu reagent [20] in 96-well microplates with some modifications. Absorbance was recorded at 765 nm and gallic acid was used to construct the standard curve. TPC of ethanol and aqueous extracts of PHAF were expressed as mg gallic acid equivalents per gram of extract on a dry weight basis.

#### 2.9.2. Total Flavonoid Content (TFC)

Total ﬂavonoid content of ethanol and aqueous extracts of PHAF was determined using the aluminium chloride method in 96-well microplates [21] with some modifications. Absorbance was recorded at 415 nm and quercetin was used to construct the standard curve. TFCs of ethanol and aqueous extracts of PHAF were expressed as mg quercetin equivalents per gram of extract on a dry weight basis.

#### 2.9.3. DPPH Radical Scavenging Activity

The DPPH (1,1-diphenyl-2-picrylhydrazyl) radical scavenging assay was performed in 96-well microplates according to the method described by Blois [22] with some modifications. Absorbance was recorded at 517 nm and trolox (12.5, 6.25, 3.12, 1.56, and 0.78 *μ*g/mL) was used to construct the standard curve. Results were expressed as trolox equivalents antioxidant capacity (mg trolox equivalents per gram of extract) on a dry weight basis.

#### 2.9.4. ABTS ^+^ Radical Scavenging Activity

The ABTS^**+**^(2,2-azino-bis(3-ethylbenzthiazoline-6-sulfonic acid) radical scavenging assay was performed in 96-well microplates according to the method described by Re [23] with some modifications. Trolox was used to construct the standard curve, and results were expressed as trolox equivalents antioxidant capacity (mg trolox equivalents per gram of extract) on a dry weight basis.

#### 2.9.5. Oxygen Radical Absorbance Capacity (ORAC)

The ORAC radical scavenging assay was performed in 96-well microplates according to the method described by Ou [24] with some modification. The assay was conducted at 37°C and pH 7.4, with a blank sample in parallel. Trolox standards (1.5 and 0.75 *μ*g/mL), fluorescein (4.8 *μ*M), and 2,2-azobis(2-amidinopropane) dihydrochloride (AAPH) (40 mg/mL) solutions were prepared in phosphate buffer (75 mM, pH 7.4) prior to use. Ethanol or aqueous extracts of PHAF were initially dissolved in DMSO. Reaction volumes of 200 *μ*L containing 100 *μ*L of 4.8 Μm fluorescein and 50 *µ*L of 15.62 and 7.81 *µ*g/mL of different concentrations of ethanol and aqueous extracts of PHAF were preincubated at 37°C for 10 minutes followed by addition of 50 *µ*L of AAPH (40 mg/mL) to each well to initiate the reaction. The plate was placed on the fluorescent microplate reader (SpectraMax-Gemini EM, Molecular Devices Inc., USA) set with excitation and emission at 494 nm and 535 nm and decay of fluorescein was recorded in 1-minute intervals for 60 minutes. Results were expressed as trolox equivalents antioxidant capacity (mg trolox equivalents per gram of extract) on a dry weight basis.

### 2.10. Statistical Analysis

Data were statistically analyzed using GraphPad Prism version 4.0. One-way analysis of variance (ANOVA) and Duncan's multiple range test were used to determine the differences among treatment means. *P* < 0.05 was regarded as significant.

## 3. Results

### 3.1. Physicochemical Parameters

Physicochemical parameters of PHAF are illustrated in Table 2.

### 3.2. Phytochemical Screening

Tannins, flavonoids, saponins, coumarins, and phenolic compounds were present in both hot water and hot-ethanol extracts. Alkaloids and cardiac glycosides were present only in aqueous extract.

### 3.3. Heavy Metals

Heavy metals such as Pb, Cd, Hg, and As were not detected in PHAF. The minimum detection levels of Pb, Cd, Hg, and As were 0.5 ppm, 0.05 ppm, 0.05 ppm, and 0.05 ppm, respectively.

### 3.4. Microbial Limits

Microbes including *Escherichia coli,* Coliforms, and *Salmonella* were not present in the PHAF. Aerobic plate counts revealed few (<10 CFU/g) yeasts and moulds (<10 CFU/g) and *Staphylococcus aureus* (<10/g).

### 3.5. Antioxidant Properties

Antioxidant properties of ethanol and aqueous extracts of PHAF powder are given in Table 3.

## 4. Discussion

In the present study, significantly more matter was extracted by ethanol than by water. Further, more extractable matter was present in hot extracts than in cold extracts (both water and ethanol). Very little acid insoluble ash was present in PHAF (Table 2), indicating the absence of silica-like impurities in the drug.

The main purpose of our study was to evaluate antioxidant activity of PHAF by using in vitro assays. Antioxidant activity has been evaluated previously for each of the seven plant species present in PHAF. Examples include antioxidant activities for *C. rotundus* [25, 26], *C. longa* [27], *A. indica* [28], *C. fistula* [29, 30], *C. fenestratum* [31], and *S. suaveolens* [32]. *G. glabra*, one of the ingredients in PHAF, is rich in glycyrrhizic acid which exhibits significant antioxidant activity [33].

According to traditional Ayurveda practice, PHAF is administered to patients in the hot water extract form. We, therefore, assayed antioxidant activities for the aqueous extract. However, hot water preparations have some drawbacks, such as unpleasant taste, and have lower stability, requiring them to be prepared freshly on each occasion. To avoid this drawback, it would be better to develop pharmaceutics such as syrups and tonics. Therefore, we also assayed the ethanol extract of PHAF, which exhibited overall higher levels of antioxidant activities than that of aqueous extract (Table 3). Ethanol extract is safe compared to other solvent (e.g., hexane, methanol, and acetone) extracts, and one of the targets of the present study is to formulate a modern drug from PHAF. Therefore, antioxidant potential of PHAF was compared with only water and ethanol extracts.

In ABTS^+^ assay, a blue/green ABTS^.+^ chromophore is generated by the oxidation of ABTS with potassium persulfate. Antioxidants which have the ability of donating hydrogen reduced the blue/green color of ABTS^+^ and can be measured spectrophotometrically at 745 nm [34]. DPPH is a stable synthetic radical and does not deteriorate in water, methanol, or ethanol [35]. In DPPH assay, presence of hydrophilic antioxidants such as proton radical scavengers or hydrogen donors, reduced the color of DPPH to straw color [36, 37] from its original deep violet color at 517 nm. Moreover, the DPPH assay determines only the hydrophilic antioxidants whereas the ABTS^.+^ assay measures both hydrophilic and lipophilic antioxidants [23].

**Figure 1:**
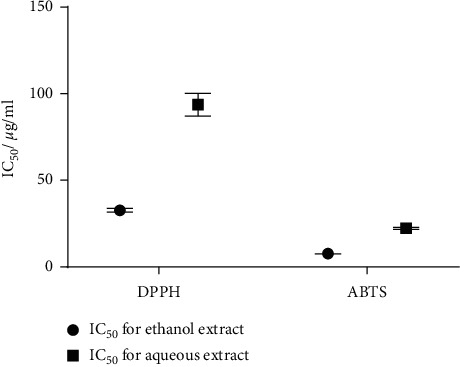
IC_50_ values of aqueous and ethanol extracts of Polyherbal Ayurvedic Formulation (PHAF) for ABTS^+^ and DPPH assays.

In the present study, IC_50_ values of ABTS^+^ (for both aqueous and ethanol extracts) were significantly lower than that of DPPH (Figure 1). This indicates that, in addition to hydrophilic antioxidants, lipophilic antioxidants play a major role in scavenging free radicals. Similar activity is exhibited in many plants such as *Toddalia asiatica, Glinus oppositifolius, Spondias pinnata*, and *Aganonerion polymorphum* [38]. In contrast, some plant extracts such as *Sonchus asper* [39] have shown more radical scavenging properties in the DPPH^.^ assay than in the ABTS^+^ assay.

The capacity of a compound to scavenge peroxyl radicals, generated by spontaneous decomposition of 2,2^,^-azobis(2-amidinopropane) dihydrochloride (AAPH), was estimated in the ORAC assay. The ORAC value is directly proportional to the degree of antioxidant power [36, 40].

Phenolic compounds and flavonoids are reported to have antioxidant and free-radical scavenging activity [41, 42]. They perform scavenging activity by stabilizing free radicals via their conjugated ring structures and hydroxyl groups [43]. The high quantity of phenolic and flavonoid contents of PHAF may contribute to its high antioxidant property. Therefore, ethanol extract of this drug has more radical scavenging activity towards both ABTS^+^ and DPPH than aqueous extract of the drug. Similar results have been shown with *Foeniculum vulgare* [44]. Among the plant metabolites, polyphenols and flavonoids are promising in governing number of bioactivities as antidiabetic, antioxidant, hepatoprotective, antimicrobial, anti-inflammatory, etc. [45, 46]. Therefore, presence of high content of polyphenols and flavonoids may play a major role in proven anti-inflammatory activity of PHAF.

The ash content is a criterion to judge the identity and purity of crude drugs [47, 48]. A high acid insoluble ash content is indicative of contamination, substitution, adulteration, or carelessness in preparing the samples for marketing. Acid-insoluble ash indicates contamination with silica, for example. earth and sand [49, 50]. Therefore, the low acid insoluble ash content in PHAF indicates high purity of the drug. Furthermore, very low levels of microbes in PHAF indicate good manufacturing practices during preparation. In addition, heavy metals such as Hg, Pb, As, and Cd were not present in PHAF. The extractable matter value is useful for the evaluation of a crude drug as it gives an idea about the nature of chemical constituents present in the drug. In addition, it is useful for the estimation of chemical constituents soluble in a particular solvent used for extraction [50].

The phytochemical constituents of many medicinal plants have been recorded by a number of researchers during the last few decades [47, 50, 51]. The most important bioactive compounds in medicinal plants are alkaloids, flavonoids, and phenolic compounds [52, 53]. The plant extracts in the present study also contained saponins, which are known to produce inhibitory effects on inflammation [54]. Furthermore, antioxidants are known to have potential against different disease conditions such as diabetes, cancer, cardiovascular disease, Alzheimer's disease, Down's syndrome, Parkinson's disease, and schizophrenia [55–59]. As PHAF is rich in antioxidants, this polyherbal formulation will be beneficial to the world.

## 5. Conclusions

We have reported the quality assessment and antioxidant properties of PHAF for the first time. PHAF has potent antioxidant properties, which may be due to the presence of high content of polyphenols and flavonoids. Even though aqueous extract of PHAF is used in traditional practice, ethanol extract can be used to formulate pharmaceuticals such as tonics and syrups due to its potent antioxidant properties.

## Figures and Tables

**Table 1:** Composition of Polyherbal Ayurvedic Formulation (PHAF).

No.	Scientific name	Family	Amount and part of the plant used
1	*Cassia fistula* Linn.	Fabaceae	Bark (10 g)
2	*Glycyrrhiza glabra* Linn.	Leguminosae	Stem (10 g)
3	*Coscinium fenestratum* (Goetgh.) Colebr.	Menispermaceae	Stem (10 g)
4	*Cyperus rotundus* Linn.	Cyperaceae	Rhizome (10 g)
5	*Curcuma longa* Linn.	Zingiberaceae	Rhizome (10 g)
6	*Azadirachta indica* A. Juss.	Meliaceae	Bark (10 g)
7	*Stereospermum suaveolens* (Roxb.) DC.	Bignoniaceae	Bark (10 g)

**Table 2:** Physicochemical parameters of Polyherbal Ayurvedic Formulation (PHAF).

Physicochemical parameters	Amount (% dry weight basis)
Moisture content	5.6 ± 0.2
Total ash content	6.5 ± 0.1
Water soluble ash content	1.4 ± 0.1
Acid insoluble ash content	0.9 ± 0.0
Hot-ethanol extractable matter	10.5 ± 4.1
Cold-ethanol extractable matter	8.4 ± 0.2
Hot-water extractable matter	7.7 ± 0.2
Cold-water extractable matter	3.9 ± 0.1

Data represented as mean ± SEM (standard error mean); *n* = 6.

**Table 3:** Antioxidant properties of Polyherbal Ayurvedic Formulation (PHAF).

Samples	Antioxidant properties				
	TPC	TFC	ORAC	DPPH	ABTS
	(mg gallic acid equivalents/g of extract)	(mg quercetin equivalents/g of extract)	(mg trolox equivalents/g of extract)	(mg trolox equivalents/g of extract)	(mg trolox equivalents/g of extract)
Ethanol extract	327.07 ± 9.65^*∗*^	224.6 ± 8.42^*∗*^	1481.44 ± 30.20^*∗*^	227.17 ± 6.16^*∗*^	577.08 ± 5.48^*∗*^
Aqueous extract	103.65 ± 4.94	76.6 ± 5.83	481.11 ± 17.30	79.50 ± 4.42	198.20 ± 4.55

Data represented as mean ± SEM (standard error mean). ^*∗*^ Significant when compared to the respective values of TPC, TFC, ORAC, DPPH, and ABTS of aqueous extract; *P* < 0.05. TPC: total polyphenol content (*n* = 6); TFC: total flavonoid content (*n* = 6); ORAC: oxygen radical absorbance capacity (*n* = 5); DPPH: 1,1-diphenyl-2-picryl-hydrazyl; ABTS: 2,2-azino-bis(3-ethylbonzothiazoline-6-sulfonic acid) diammonium salt (*n* = 4 each).

## Data Availability

All the data obtained and materials analyzed in this research are available from the corresponding author upon request and also can be accessed from Ph.D. thesis of the corresponding author deposited at the University of Kelaniya, Sri Lanka.
